# A Light‐Triggered Synthetic Nanopore for Controlling Molecular Transport Across Biological Membranes

**DOI:** 10.1002/anie.202210886

**Published:** 2022-11-28

**Authors:** Daniel Offenbartl‐Stiegert, Alexia Rottensteiner, Adam Dorey, Stefan Howorka

**Affiliations:** ^1^ Department of Chemistry Institute for Structural and Molecular Biology University College London WC1H0AJ London UK

**Keywords:** DNA Nanotechnology, Membranes, Nanopores, Photoswitch

## Abstract

Controlling biological molecular processes with light is of interest in biological research and biomedicine, as light allows precise and selective activation in a non‐invasive and non‐toxic manner. A molecular process benefitting from light control is the transport of cargo across biological membranes, which is conventionally achieved by membrane‐puncturing barrel‐shaped nanopores. Yet, there is also considerable gain in constructing more complex gated pores. Here, we pioneer a synthetic light‐gated nanostructure which regulates transport across membranes via a controllable lid. The light‐triggered nanopore is self‐assembled from six pore‐forming DNA strands and a lid strand carrying light‐switchable azobenzene molecules. Exposure to light opens the pore to allow small‐molecule transport across membranes. Our light‐triggered pore advances biomimetic chemistry and DNA nanotechnology and may be used in biotechnology, biosensing, targeted drug release, or synthetic cells.

## Graphical abstract

A synthetic light‐gated nanopore controls transport of molecules across bilayer membranes. The pore self‐assembles from seven DNA strands including one lid strand carrying light‐switchable azobenzene chromophores. The gated pore may be used for research, biosensing, and targeted drug release.

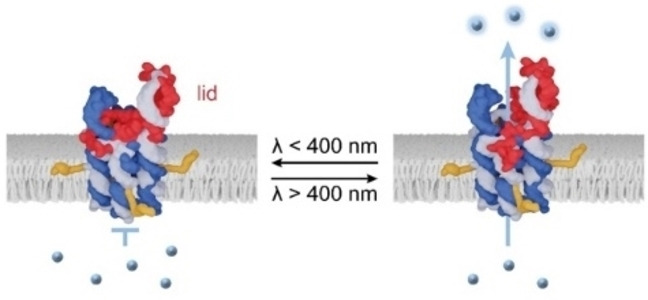

## Introduction

Controlling biomolecular processes with light is a powerful principle widely used in biology and increasingly enhanced via synthetic means.^[1]^ In biology, light response is mediated via protein photosensors[^2^, ^3^] that undergo triggered conformational changes to activate molecular interactions^[3]^ and gene expression^[2]^ for applications in biological research and medicine.^[4]^ Light control via synthetic routes can expand the scope of biology by making previously non‐responsive biomolecules photo‐activatable,[^5^, ^6^] thereby opening up new applications.^[7]^ In one popular approach, photocages are covalently attached and then light‐removed to trigger protein dimerisation,^[8]^ protein phosphorylation,^[9]^ DNA methylation,^[10]^ and release of bioactive molecules.^[11]^

One molecular process that benefits from light control is the transport of molecular cargo across lipid membranes. Transport across membranes is often mediated by bilayer‐embedded nanopores. These constitutively open barrel‐like structures are used in next‐generation portable DNA sequencing and biosensing,[^12^, ^13^, ^14^, ^15^, ^16^, ^17^, ^18^, ^19^, ^20^, ^21^, ^22^, ^23^, ^24^] where individual analyte molecules pass through the channel and cause electrical signatures.[^23^, ^24^, ^25^, ^26^, ^27^, ^28^] Membrane pores with a light‐gated valve‐like function are also of interest and advance biosensing,^[29]^ drug delivery,^[30]^ and the formation of synthetic organelles.^[31]^

In biology, light‐controlled transport across membranes is mediated by dedicated membrane proteins. The light‐gated ion channels or channelrhodopsins perforate membranes and use a chromodomain to sense light and trigger channel opening or closing.[^32^, ^33^] The light control can be harnessed to precisely control channel activity for neurological research in a minimally invasive and remote manner.[^34^, ^35^, ^36^]

Semi‐synthetic or entirely synthetic light‐gated nanopores can offer more design flexibility and broader functional scope. In one semi‐synthetic route, the essential trigger wavelength is tuned by modifying a biological non‐gated nanopore with synthetic light‐sensitive actuators including azobenzene[^37^, ^38^, ^39^] to reversibly open the resulting nanovalve for controlled release from membrane containers^[40]^ or timed pore assembly.^[41]^ By using existing biological nanopores, semi‐synthetic approaches cannot drastically alter the channel width even though this helps transport large bioactive cargo. De novo design of narrow protein channels is established,[^42^, ^43^, ^44^, ^45^] but building wide channels as well as incorporating light activation and actuated channel opening is currently out of reach given the difficulties in predicting the folding of complex proteins.

DNA is an alternative material for highly predictable design and may help create synthetic light‐gated nanopores. Building with DNA[^46^, ^47^] takes advantage of the precise self‐assembly via base pairing,^[48]^ computer‐aided design software,^[49]^ well‐known structural duplex parameters, and versatile oligonucleotide synthesis.[^50^, ^51^] Indeed, synthetic membrane channels have been made with DNA[^52^, ^53^, ^54^, ^55^, ^56^, ^57^] also to open in response to effectors such as oligonucleotides,[^58^, ^59^] protein^[60]^ and temperature.^[61]^ However, light‐gated DNA pores have yet to be realised. As DNA is not inherently photo‐tuneable, covalent coupling of a chemical chromophore could deliver the desired functional nanostructure.

Here we present a DNA‐made light‐controlled nanopore (LP) which, upon irradiation, opens for transporting molecular cargo across membranes in a non‐invasive manner. Light‐responsiveness is achieved with an azobenzene‐modified^[62]^ DNA lid which photo‐reversibly attaches to the entrance of the LP channel and thereby regulates capacity for transport. The LP advances the design of previous, gated pores by allowing light‐mediated activation of the opening mechanism. We envision our synthetic light‐gated nanopore to be exploited in neurobiological research, biosensing, targeted drug delivery, or the construction of synthetic cells.

## Results and Discussion

**Figure 1:**
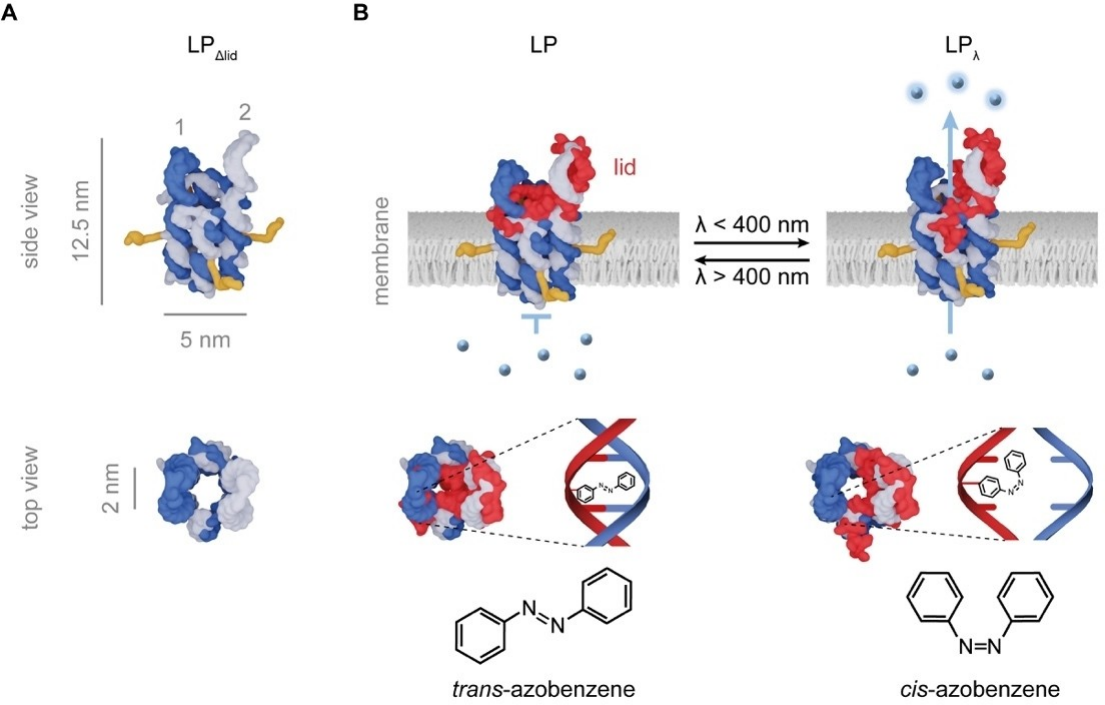
Design and functional principle of the light‐controlled nanopore LP. A) Model of pore variant LP_Δlid_ without a lid in the side and top view with indicated pore dimensions. The pore is composed of 6 DNA strands (dark and light blue). Attached cholesterol tags (orange) facilitate membrane insertion of the pore, as shown in B. The two hinge regions are termed 1 and 2. B) Scheme on the reversible light‐gated opening of membrane‐inserted LP which carries a lid strand (red) modified with photosensitive azobenzene moieties. In the closed state of LP (left panel), transport of small‐molecule cargo (blue spheres) is blocked by the lid strand bound across the pore entrance. Irradiation with light <400 nm leads to partial dissociation of the lid and channel opening to LP_λ_ to allow the transport of cargo (right panel). The zoom‐ins show part of a duplex between hinge 1 (dark blue) and the azobenzene‐modified lid strand (red) in the closed channel and ‐after illumination‐ the dissociated DNA duplex of the opened channel. The *trans* azobenzene is a flat molecule and intercalates between the base pairs to stabilise the duplex while the *cis* isomer is buckled, does not fit between base pairs, and leads to dissociation of the duplex. Details of the binding pattern of the lid strand in the open and closed pore are provided in Figure S1.

The design of LP comprises a barrel‐shaped nanopore[^60^, ^61^, ^63^] with a light‐controlled lid. The nanopore is formed by 6 DNA strands which assemble into 6 double helices that are interlinked by hairpins and arranged in hexagonal fashion (Figure 1A, Table S1, S2). The nanopore measures up to 12.5 nm in height and 5 nm in outer diameter and encloses a 2 nm‐wide channel lumen (Figure 1A). Two of the pore's duplexes feature elongated unpaired sequence lobes, termed “hinges”, for hybridisation to the lid strand (Figure 1A, B, Figure S1). The hinges are numbered 1 and 2 (Figure 1A). In LP's closed state, the lid strand is bound to the two hinges and thereby blocks the channel entrance for the transport of molecular cargo (Figure 1B, left panel).

To function as a light‐sensitive nanovalve, LP's lid strand is equipped with photo‐switchable azobenzene moieties (Figure 1B, Figure S1). Azobenzene is a well‐studied chromophore which undergoes a light‐switchable and reversible *cis*‐*trans* isomerisation (Figure 1B).^[64]^ The light‐tuneable isomerisation has been exploited for controlling biomolecular processes[^65^, ^66^, ^67^] and can be triggered by irradiation at wavlengths, λ, < 400 nm to switch the *trans* to the *cis* isomer, or to achieve the reverse by illumination at λ > 400 nm whereby the wavelengths are the isomers’ absorption maxima. When incorporated into a DNA strand, the *trans* isomer allows for duplex formation as the flat chromophore can intercalate into neighbouring base pairs to stabilise the duplex (Figure 1B). By contrast, irradiation with UV light at wavelength < 400 nm yields the buckled azobenzene *cis* isomer which cannot intercalate and destabilises the duplex by steric interactions (Figure 1B).[^65^, ^66^, ^67^]

We exploited the photo‐triggered duplex disassembly to build the light‐gated nanopore. In LP, the lid strand carries the azobenzene moieties at the terminally flanking sequences which hybridise to hinge 1 (Figure 1B, Figure S1) when azobenzene is in the *trans* isomeric form. The unmodified sequence centre of the lid strand hybridises to hinge 2 (Figure 1B, Figure S1). The lid strand bound to both hinges constitutes the closed LP state. Irradiation‐triggered isomerisation of azobenzene from *trans* to *cis* selectively breaks the DNA duplexes between the lid and pore at hinge 1 to form the open LP_λ_ pore (Figure 1B). The lid remains hybridised to hinge 2 given the high melting temperatures of the duplexes at 70 °C. The high thermal stability is a result of rationally designedlonger hinge sequences with a higher GC content. By comparison, the melting temperatures of hinge 1 duplexes were designed to be at 45 °C. We note that the DNA lid could also be designed to bind in another pattern to hinges 1 and 2. In one alternative pattern, one terminus of the lid strand stays bound to hinge 1 while the other half can be dissociated in the presence of a trigger.[^60^, ^61^] Applied to the light‐gated pore, this binding pattern would require a lid strand with many azobenzene modifications clustered within one strand segment. As this is synthetically challenging, our present LP design employs azobenzene modifications distributed at the two lid termini (Figure 1B). This pattern also improves the lid strand's aqueous solubility required for DNA nanopore assembly. As other chemical modification, LP contains four cholesterol anchors (Figure 1, orange) to embed the pore into a bilayer membrane.

**Figure 2:**
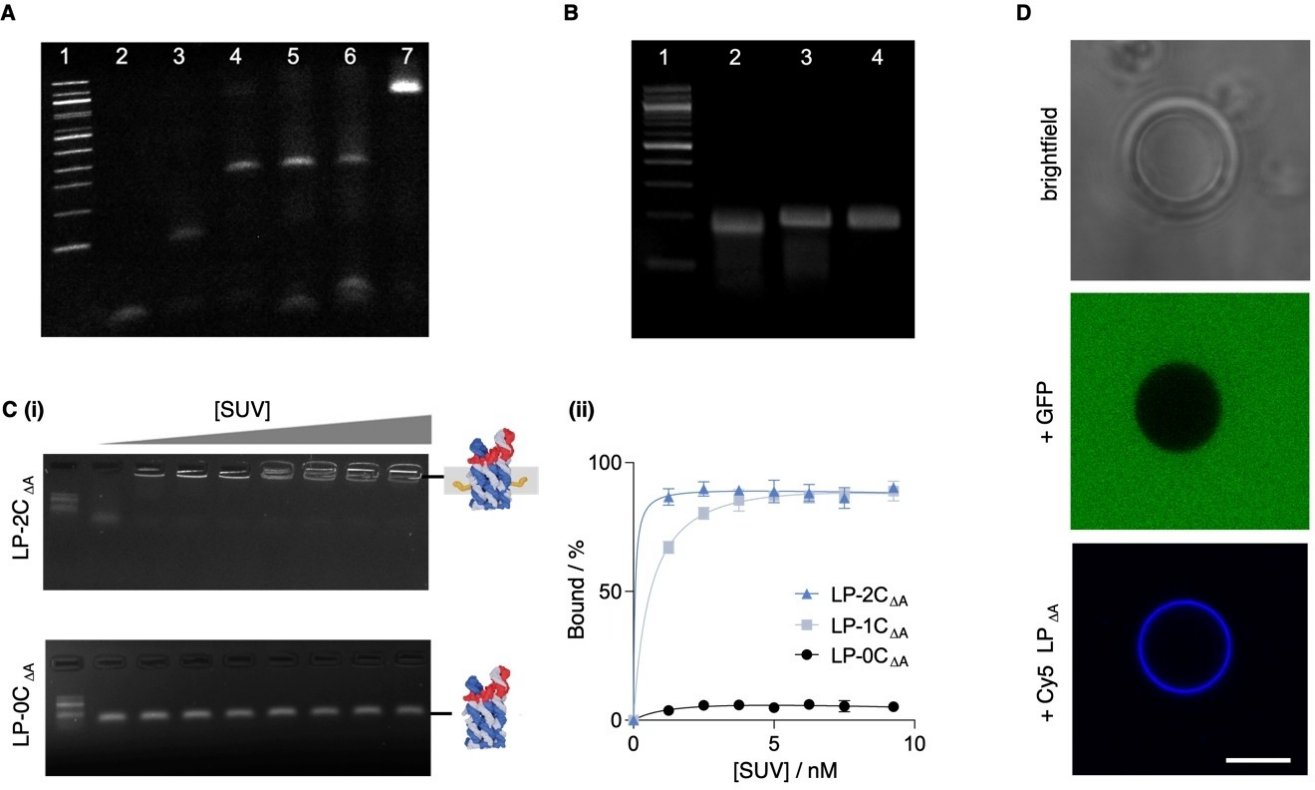
Assembly and membrane binding of LP pore variants. A) SDS PAGE analysis of self‐assembled variant LP‐0C_Δlid_ without a lid and other assemblies containing from two to six of the pore strands. 100 bp ladder (lane 1), pore strand S1 (lane 2), strands S1 and S2 (lane 3), strands S1 to S3 (lane 4), S1 to S4 (lane 5), S1 to S5 (lane 6), LP‐0C_Δlid_ composed of strands S1 to S6 but without the lid strand (lane 7). B) PAGE gel shift analysis confirming successful incorporation of the lid. 100 bp ladder (lane 1), LP‐0C_Δlid_ (lane 2), LP‐0C_ΔA_ carrying a non‐modified lid strand (lane 3), LP‐0C with an azobenzene‐modified lid strand (lane 4). C‐i) PAGE analysis on the binding of LP‐2C_ΔA_ and LP‐1C_ΔA_ to SUV membranes added at increasing concentrations from 0 nM to 10 nM. The 100 bp ladder is at the leftmost lane. C‐ii) Quantitative analysis on LP membrane binding results of panel i. The normalised intensities of gel bands for SUV‐bound LP_ΔA_ with up to 2 cholesterols are plotted against SUV concentration. The data were derived from three independent experiments. D) Brightfield (top) and confocal fluorescence microscopy images of a GUV embedded in a solution of green fluorescent protein (middle) and after incubation with Cy5‐LP_ΔA_. Scalebar, 20 μm.

The light‐gated nanopore was self‐assembled by annealing an equimolar mixture of six pore DNA strands and the azobenzene‐modified lid strand. In initial experiments, a pore variant without cholesterol anchors was prepared. Successful assembly of the nanopore was confirmed by a single band in agarose gel electrophoresis and polyacrylamide gel electrophoresis (PAGE) (Figure 2A, lane 7; Figure 2B, lane 4; Figure S2). Replacing the modified lid strand with a non‐modified version did not alter the pore's band migration (Figure 2B, lane 3; Figure S2). The nanopore was also formed without the lid strand (Figure 2B, lane 2) and migrated faster as a smaller structure than the larger, lidded nanopore (Figure 2B, lanes 3 and 4). Omitting one or more of the pore strands led to incomplete assembly, smaller structures, and faster‐migrating bands (Figure 2A, lanes 2 to 6). Successful assembly of the complete lidded nanopore was corroborated by canDO simulations (Figure S3).

To achieve membrane binding of the light‐gated nanopore, up to four of the pore strands were modified with cholesterol membrane anchors. These studies were conducted with variants without any azobenzene (denoted by _ΔA_) as the modification is not required for binding to membranes. The corresponding nanopores with zero, one, two and four cholesterol tags, LP‐0C_ΔA_, LP‐1C_ΔA_, LP‐2C_ΔA_ and LP_ΔA_, respectively, were assembled and subsequently assessed via PAGE; LP_ΔA_ does not feature 4C in its name for reasons of simplicity. A band upshift characteristic for cholesterol‐modified nanostructures^[52]^ confirmed the successful formation of the cholesterol‐tagged DNA nanopores (Figure S4). LP‐1C_ΔA_ was expected to only tether to the membrane while LP‐2C_ΔA_ and LP_ΔΑ_ were assumed to span lipid bilayers (Figure S5).^[68]^

Membrane binding of the cholesterol‐tagged nanopore was demonstrated with small unilamellar membrane vesicles (SUVs) composed of phospholipid dipalmitoyl phosphatidylcholine (DOPC)(Figure S6). Pores without cholesterol tags (LP‐0C_ΔA_ ), and with one (LP‐1C_ΔA_) and two modifications (LP‐2C_ΔA_) were used. Incubating the cholesterol‐carrying light‐gated pores with SUVs led to an electrophoretic gel band upshift indicative of successful binding of DNA pores to SUVs. The bands are upshifted as the DNA‐vesicle complexes are too large to migrate into the gel matrix and remain in the gel loading well (Figure 2C‐i, Figure S7). Incubation with SUVs at increasing concentrations caused higher proportions of LP‐1C_ΔA_ and LP‐2C_ΔA_ to bind to membranes, as illustrated by quantitative analysis of gel band intensities (Figure 2C‐ii). Negative LP‐0C_ΔA_ only showed minimal binding to membrane vesicles (Figure 2C‐ii), as expected for the absence of a lipid anchor.

Binding of cholesterol‐tagged nanopore to vesicles was also investigated by confocal fluorescence microscopy. To facilitate their detection, the pores were equipped with a Cy5 fluorophore. The fluorophore‐modified nanopores were incubated with giant unilamellar vesicles (GUVs) composed of DOPC. Green fluorescent protein (GFP) was added to a suspension of GUVs to display the vesicles’ outline in microscopic analysis (Figure 2D, middle panel). Confocal imaging in the Cy5 fluorescence channel showed a halo‐like ring around the vesicles, confirming membrane binding of Cy5‐labelled LP_ΔA_ (Figure 2D, bottom panel). Similar halo‐like binding was also found for Cy5‐LP‐2C_ΔA_ and Cy5‐LP‐1C_ΔA_ but not for negative control Cy5‐LP‐0C_ΔA_ lacking the cholesterol tag (Figure S8–S10).

To probe membrane insertion of the cholesterol‐tagged pore, UV melting profiles were obtained. Membrane insertion is known to increase pore stability as previously demonstrated by higher melting temperatures (*T*_m_).[^54^, ^63^, ^69^] The *T*_m_ of LP_ΔA_ was increased by 6.2±0.6 °C in the presence of vesicles composed of diphytanoyl phosphatidylcholine (DPhPC) (Figure S11), suggesting membrane poration. In comparison, the *T*_m_ increased for LP‐2C_ΔA_ and LP‐1C_ΔA_ were 6.1±1.0 °C and 5.5±0.6 °C indicating spanning and possibly partial pore insertion or tethering, respectively (Figure S11). The *T*_m_ for negative control LP‐0C_ΔA_ was unaffected by the presence of vesicles (Figure S11). As a minor observation, increasing numbers of cholesterol tags led ‐in the absence of SUVs‐ to a slight increase of *T*_m_ (Figure S11), which may be due to hydrophobic interactions between pores.^[70]^ Any extent of oligomerisation must be small as gel electrophoretic analysis suggests mostly monomeric nanopores (Figure S4).

**Figure 3:**
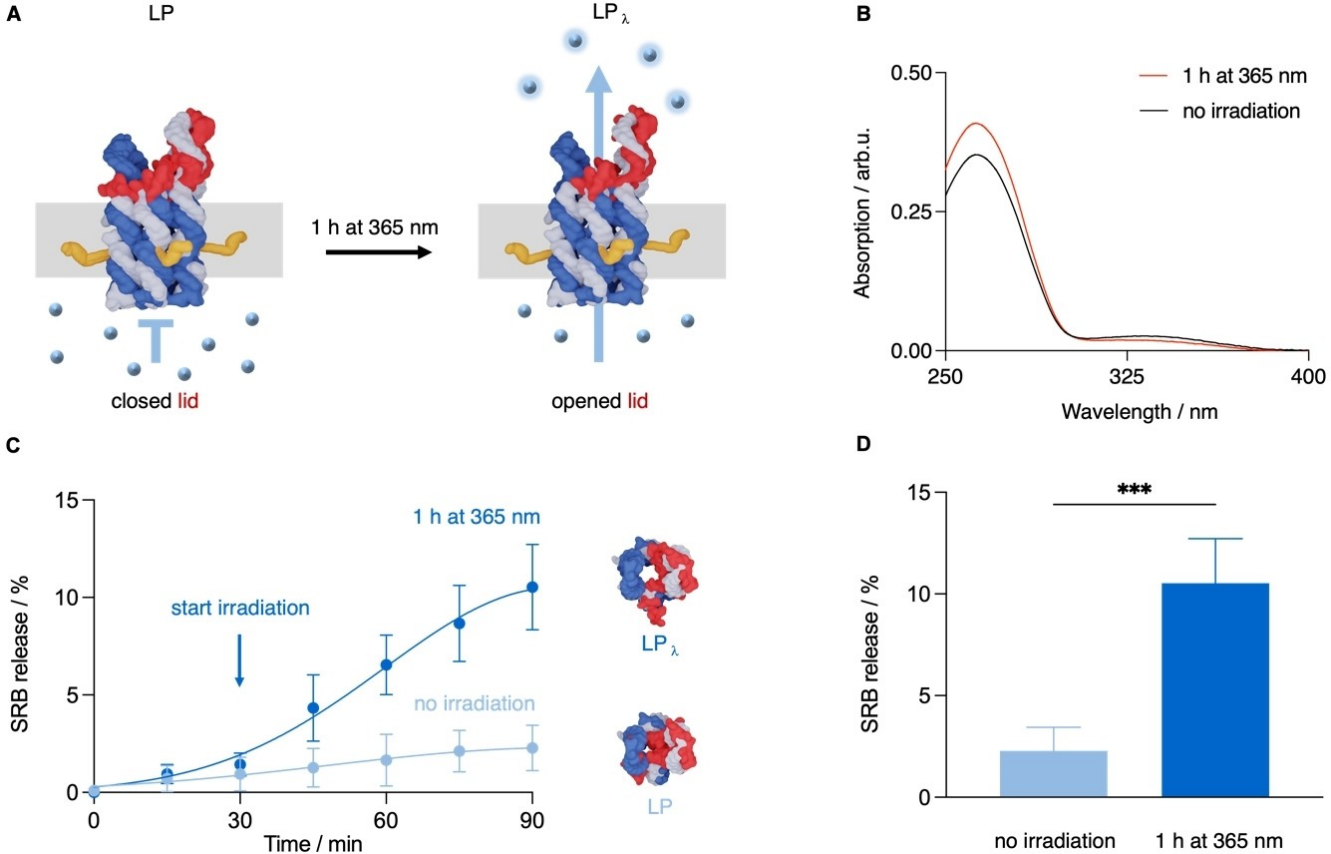
Light‐gated small‐molecule transport through LP. A) Model of membrane‐inserted LP in the closed state (left) where the lid blocks the channel entrance as well as molecular flux, and the open state LP_λ_ (right) after irradiation at *λ*_max_=365 nm leading to partial dissociation of the lid and flux of small‐molecule dye SRB. B) UV/Vis absorption spectrum of azobenzene‐modified lid strand before and after irradiation at *λ*_max_=365 nm at an intensity of 145 μW cm^−2^. C) Kinetic analysis of SRB efflux from SUVs with inserted LP without irradiation and upon light exposure at 365 nm. Irradiation was started (arrow) 30 min after incubating LP with SUVs. The datapoints±SD were taken every 15 min and summarise five independent experiments. A scheme illustrating the experimental design of the flux assay is shown in Figure S16. The experiments were conducted with nanopores carrying four cholesterol tags. For reasons of visual clarity, the four cholesterol anchors are not shown in the top‐down view of the nanopores. D) Analysis of dye release after 60 min as shown in panel C. Unpaired t‐test with *** *P* value of 0.0003.

After having established the membrane‐spanning nature of the pore, we probed its lighted‐controlled opening and the associated activation of cargo transport across the transmembrane channel (Figure 3A). The light‐induced switching of the pore's lid from the *trans*‐azobenzene to the *cis* isomer form (Figure 3A) was first demonstrated with UV/Vis absorption spectroscopy. Upon triggering isomerisation by irradiation at 365 nm, the absorption of azobenzene at 340 nm decreased but increased at 260 (Figure 3B, Figure S12–S14). Both features are characteristic for the *cis* isomer.^[62]^ The reversible switching from the *cis* back to the *trans* isomer upon irradiation with visible light was also shown via corresponding changes in the absorption spectrum (Figure S12–14). No difference between the *cis* and *trans* nanopore forms was visible by gel shift analysis (Figure S15), as expected for the small change in structure. The gel results also indicate pore stability and lid attachment upon prolonged UV irradiation and partial lid opening.

The light‐controlled opening of LP for transport across the membrane was probed using small‐molecule fluorophore sulforhodamine B (SRB). In the assay, SRB was encapsulated within SUV vesicles at self‐quenching concentrations (Figure 3A, Figure S16). No dye efflux was expected for LP with a fully hybridised lid carrying *trans*‐azobenzene (Figure 3A). Indeed, addition of non‐irradiated LP to SRB‐filled SUVs retained the dye inside the vesicle with only minimal leakage (Figure S17). Successful dye efflux and increase of fluorophore emission due to reversal of quenching was established with a constitutively open LP_Δlid_ carrying four cholesterol tags (Figure S17). Our flux measurements do not distinguish between pores in the upright membrane‐inserted orientation (Figure 3A) or with the lid facing down. Both orientations should facilitate light‐controlled lid opening.

We analysed the kinetics of small‐molecule transport upon light‐gated opening of LP. The DNA nanopore was mixed with SRB‐filled SUVs for 30 min before being irradiated with 365 nm light for 1 h (Figure S16). The SRB efflux was monitored at intervals of 15 min. Irradiation of LP led to a fluorophore release with an endpoint at 10.5±2.2 % (Figure 3C,D). This is a 5‐fold increase to the 2.3±1.2 % background release for LP with closed lid (Figure 3C, D). The percentage values are normalised to maximum fluorophore release achieved upon rupturing vesicles with a detergent. The reference pore α ‐hemolysin featured a flux of 28.6±5.9 % (Figure S18), in line with the fast transport properties of the protein pore.^[53]^ Further dye flux experiments with either LP_ΔA_ lacking azobenzene or vesicles without pores confirmed that prolonged UV irradiation did not affect pore stability or leakage of the closed pore, respectively (Figure S19). Furthermore, light‐gated transport across membrane‐inserted LP was found when probing SRB influx into GUVs as determined via fluorescence microscopy (Figure S20). The reversibility of lid opening was confirmed by stopping the UV irradiation of LP after 30 min and starting exposure to visible light (>400 nm) which caused the plateauing of SRB release (Figure S21). We note that the difference in flux between the open and closed DNA pore is smaller than for other gated DNA nanopores.[^60^, ^61^] This might be due to partial blockade of the pore in the open state as a consequence of the different binding patterns of the lid strand to hinges 1 and 2 of the DNA pore (Figure 1C). Alternatively, the isomerisation from *trans* to *cis‐*azobenzene might be incomplete.

**Figure 4:**
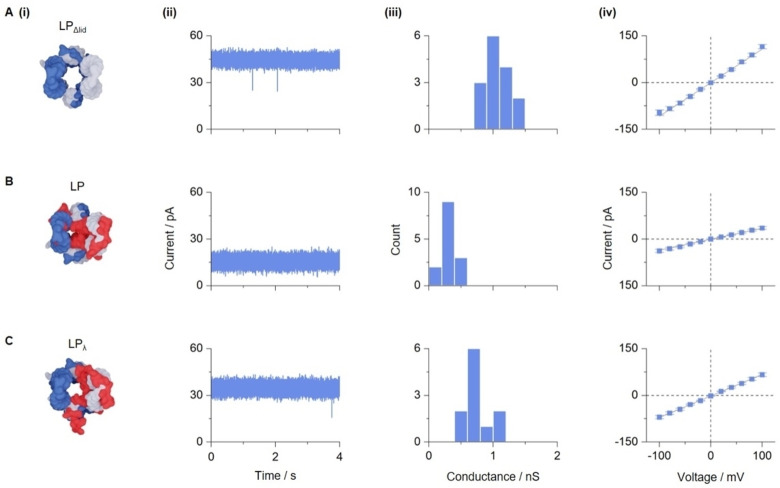
Single‐channel current recordings of lid‐free LP_Δlid_ and LP with an azobenzene‐modified lid strand in either the closed state or light‐opened state. Analysis using single‐channel current recordings of A) LP_Δlid_, B) LP without—irradiation, and C) LP_λ_ after irradiation for 30 min at 365 nm with an intensity of 145 μW cm^−2^ (C). Panels show i) the top view of DNA nanopores, ii) representative single‐current traces recorded at +50 mV relative to the *cis* chamber, iii) conductance histograms obtained at +20 mV, and iv) average current–voltage graphs (±SEM) for voltages ranging from −100 mV to +100 mV in 20 mV steps. All pores carried four cholesterol tags. For the recordings, the nanopores were mixed with mild detergent octyl polyoxyethylene and then added to the *cis* chamber for membrane insertion. The recordings were acquired in 1 M KCl, 10 mM HEPES, pH 7.4. Data in iii and iv are from 15, 14 and 11 individual insertions of pores LP_Δlid_, LP without irradiation and LP_λ_ with irradiation, respectively. The experiments were conducted with nanopores carrying four cholesterol tags. For reasons of visual clarity, the four cholesterol anchors are not shown in the top‐down view of the nanopores in panel i.

To explore the structural dynamic changes of the nanopore upon light‐controlled opening, single‐channel current recordings were conducted. Electrical recordings have previously been successfully used to distinguish different conformational states of DNA nanopores.[^58^, ^60^] Firstly, LP_Δlid_ was investigated to determine baseline currents for the pore without the lid (Figure 4A‐i). The addition of LP_Δlid_ to a planar lipid bilayer led to distinct increases of current, as shown by a representative trace with 44 pA at +50 mV using an Orbit Mini device with *trans* grounding and a *cis* working electrode (Figure 4A‐ii). The current was monitored as a function of voltage for 15 independent pore insertions. The mean conductance for LP_Δlid_ was found to be 1.10±0.06 nS (*n*=15) (Figure 4A‐iv). This value is slightly below previous reports for similar DNA nanopores.[^21^, ^53^] Possibly, our pore's long hinge regions for the lid may partially block the lumen or otherwise impede ion transport. The ohmic current–voltage dependence of LP_Δlid_ was linear and in line with comparable DNA pores (Figure 4A‐iii)^,[58]^.^[54]^ By contrast, non‐irradiated LP with the closed lid led to considerably smaller single‐pore currents, as illustrated by a trace of 15 pA at +50 mV (Figure 4B‐ii). This reduction in current underlines the ability of the closed lid to block the entrance of the channel (Figure 4B‐i). Closed LP had a mean conductance of 0.32±0.04 nS (*n*=14) (Figure 4B‐iii, iv) which is lower than pores without the lid. Small residual currents as opposed to no currents likely stem from small gaps between the lid and the barrel‐pore (Figure 4B‐i).

Analysis of light‐opened LP_λ_ established the expected higher current, as shown by a representative trace with 35 pA at +50 mV (Figure 4C‐ii). The mean conductance for the light‐opened pore was 0.75±0.06 nS (*n*=11) (Figure 4C‐iii, iv) which is an increase compared to the non‐irradiated closed pore and confirms the light‐gated opening of irradiated LP_λ_ (Figure 4C‐i). The conductance at 0.75±0.06 nS is lower than LP_Δlid_ as the open lid of irradiated LP_λ_ is still bound to the pore and partially blocks the channel lumen (Figure 4C‐i). The percentage of pores that remained in the closed state after irradiation was calculated by the extent of overlap of the conductance histograms for LP and LP_λ_. These calculations yielded an overlap of 18 % which is a boundary for any incomplete change from *trans* to *cis*‐azobenzene. The overlap may also reflect the incomplete separation of the two pore types’ conductances. Further control experiments showed that irradiation‐dependent lid opening is only possible when azobenzene is present in the lid strand. In the corroborating measurements, the conductance of LP_ΔA_ before (Figure S22) and after irradiation (Figure S23) did not result in an increase in pore conductance in comparison to non‐irradiated pores.

## Conclusion

This study has described the first completely synthetic light‐gated nanopore capable of controlling molecular transport across biological membranes. Previous synthetic channels made of organic molecules or peptides were not light‐gated,[^71^, ^72^] while DNA channels were either constitutively open[^52^, ^53^, ^54^, ^55^, ^56^, ^57^] or featured a gating principle triggered by oligonucleotides,[^58^, ^59^] protein,^[60]^ and elevated temperature.^[61]^ The latter pores highlighted the strength of DNA for the rational design of gated membrane channels. To achieve the desired light‐responsiveness, this report has synergistically combined DNA nanotechnology with photochemistry to create a DNA nanopore equipped with an azobenzene‐based light switch. The entirely synthetic approach offers advantages over semisynthetic or biological routes. One advantage is the greater design scope as DNA building blocks can easily create wider membrane channels for the transport of larger biologically active cargo including proteins,[^21^, ^59^] something which would be difficult to achieve with protein‐based channels. Using DNA also advances in design of the nanomechanical gate and associated kinetic opening or closing parameters.^[51]^ A specific advantage of chemical as opposed to biological light‐switches is the ease of engineering a response to longer wavelengths^[73]^ or tune the number and position of light‐sensitive molecules within the channel. A potential disadvantage of purely synthetic channels is that they cannot be generated within biological cells but have to be premade and then added to cells. Within these boundaries, there is a wide range of exciting applications for synthetic light‐gated nanopores in research, controlled drug release, biosensing as well as the construction of light‐triggered artificial signalling networks in cell‐like containers.^[74]^ Furthermore, the light‐gated pore can be employed for studying the transport of nucleic acids, such as miRNA, where inorganic particles are conventionally used for nucleic acid delivery.[^75^, ^76^]

In conclusion, the light‐controlled nanopore advances biomimetic chemistry, DNA nanotechnology, and nanopore design. It may also inspire more light‐controlled nanopores and other photo‐gated membrane‐spanning DNA nanostructures.

## Conflict of interest

The authors declare no conflict of interest.

1

## Supporting information

As a service to our authors and readers, this journal provides supporting information supplied by the authors. Such materials are peer reviewed and may be re‐organized for online delivery, but are not copy‐edited or typeset. Technical support issues arising from supporting information (other than missing files) should be addressed to the authors.

Supporting Information

## Data Availability

The data that support the findings of this study are available from the corresponding author upon reasonable request.
