# Supplementary material for: A Light‐Triggered Synthetic Nanopore for Controlling Molecular Transport Across Biological Membranes
Source: Angew Chem Int Ed Engl. 2022 Nov 28;61(52):e202210886. doi: 10.1002/anie.202210886 (PMC10098474; doi:10.1002/anie.202210886)
Supplement: Supplementary file 1 — Supporting Information [file ANIE-61-0-s001.pdf]

Angewandte Chemie
Eine Zeitschrift der Gesellschaft Deutscher Chemiker

Supporting Information

A Light-Triggered Synthetic Nanopore for Controlling Molecular Transport Across Biological Membranes

*D. Offenbartl-Stiegert, A. Rottensteiner, A. Dorey, S. Howorka**

1. Experimental section

1.1. Materials

Unmodified, cholesterol-tagged, fluorescent-labelled, and azobenezene-modified oligonucleotides were purchased from Integrated DNA Technologies with HPLC purification. Lipids 1,2-diphytanoyl-sn-glycero-3-phosphocholine (DPhPC) and 1-palmitoyl-2-oleoyl-glycero-3-phosphocholine (POPC) were purchased in chloroform solution from Avanti Polar Lipids. All other reagents were bought from Sigma Aldrich, unless stated otherwise.

1.2. Assembly of LP

All DNA sequences and 2D connectivity maps of the DNA nanopores are provided in Table S1 and Figure S1, respectively. For pore assembly, equimolar mixtures of DNA oligonucleotides (1 µL each, stock concentration of 100 µM) were diluted in 16 mM $MgCl_2$ to a final volume of 100 µL. Folding was achieved with a BioRad PCR thermocycler using heating to 95 °C for 10 min, cooling for 0.5 °C per min to 60 °C, followed by cooling at 1°C per min to 20 °C.

1.3. PAGE

Folded LP nanopores and variant pores were analysed with commercially procured 10% and 4-20% polyacrylamide gels. For pores lacking cholesterol tags, the gel buffer was 1 x Tris-Borate-EDTA buffer (TBE, from 10 x TBE buffer,89 mM Tris-borate and 2 mM EDTA, pH 8.3)(ThermoFisher scientific) and for pores with cholesterol anchors the buffer was 1 x Tris/ Glycine/ Sodium Dodecyl Sulphate (1x TGS, from 10 x TGS (25 mM Tris, 192 mM glycine and 0.1% SDS, pH 8.6)(BioRad). After equilibrating the gels (30 min at 100 V and 30 min at 0 V), the samples were loaded with gel loading dye using a 100 bp marker as a reference standard. The gels were run for a desired time at 100 V. Afterwards, the gels were washed with water and stained with ethidium bromide. The bands were visualised with UV illumination (Azure Biosystems c Series Imagine System).

1.4. Agarose gel electrophoresis

Folded LP and variant nanopores were analysed with agarose gels (1-3%) in 0.5 x TBE supplemented with 10 mM $MgCl_2$, and ethidium bromide at a dilution of 1:30,000 of the stock solution (10 mg/mL in H_2O). The DNA samples were loaded with gel loading dye using a 1 kb/100 bp marker as reference.[1] The gel was run for a desired time at 65 V, and gel bands were visualised with UV illumination (Azure Biosystems c Series Imagine System).

1.5. Formation of small unilamellar vesicles (SUVs)

A solution of POPC or DPhPC in chloroform (100 µL, 10 mg/ mL) was added to a 2 mL screw-top vial, and the solvent evaporated using argon gas under reduced pressure while rotating the vial to yield a dry lipid film. The lipid film was resuspended in 1 X Phosphate buffered saline buffer (PBS; 137 mM NaCl, 2.7 mM KCl, 10 mM Na_2HPO_4, 1.8 mM KH_2PO_4, pH 7.4) (1 mL) and vortexed to fully dissolve the lipid. After 30 min of sonication, the vesicles were extruded 25 times through an Avanti Mini-Extruder with a size-specific filter (200 nm) to yield vesicles with the set diameter. The diameter of SUVs was confirmed by dynamic light scattering (DLS) with the Zetasizer Nano S Malvern.

1.6. Binding of DNA nanopores to SUVs

The binding of LP to vesicles was monitored by incubating DNA nanopores with POPC SUVs (1:1 volume ratio, LP concentration of 1 µM) at different lipid concentrations (2-200 µM). The mixture was incubated for 30 min at RT and analysed via a 1.5% agarose gel in 0.5 TBE supplemented with 10 mM $MgCl_2$ and EthBr.[1] The gel was run for 90 min at 65 V and 4°C, and bands were visualised with UV illumination (Azure Biosystems c Series Imagine System).

1.7. Formation of giant unilamellar vesicles (GUVs)

POPC in chloroform (36 µL, 100 µM lipid concentration) was added within a rubber ring on an indium tin oxide (ITO) coated glass slide and allowed to dry. To the dried lipid film, 200 µL of 400 mM sucrose was added, another ITO slide was placed on top, and the assembly was inserted into Nanion Vesicle Prep Pro. GUVs were formed via electroformation using the conditions of 10 Hz, 4V amplitude, 1 min rise, 100 min main, and 10 min fall.

1.8. Confocal microscopy

POPC GUVs (10 µL, in 400 mM sucrose) were mixed with 1 x PBS buffer (100 µl) and a solution of green fluorescent protein (GFP,5 µL, 100 µM) on a fluorodish, and images were obtained with a SPEinv LEICA microscope. Cy5-$LP_{\Delta A}$ (10 µL, 1 µM) was added, and images were obtained after 30 min using brightfield, 395 nm (GFP) and 670 nm (Cy5) lasers.

1.9. Melting point analysis via UV-Vis spectroscopy

The melting temperatures of LP variants carrying up to 4 cholesterol tags were determined using a Varian Cary 300 Bio UV–Vis spectrophotometer with a Peltier element and a quartz cuvette with a 1 cm path length. Changes in absorbance at 260 nm of 0.1 µM of LP were monitored by heating at a rate of 1 °C per min to yield a full melting profile from 20°C to 80°C.[1]

1.10. Formation of fluorophore-filled SUVs

A solution of DPhPC in chloroform (100 µL, 10 mg/mL) was added to a 2 mL-screw top vial, and the solvent evaporated using argon gas under reduced pressure while rotating the vial to yield a dry lipid film. The film was resuspended in 1 mL of fluorescent buffer (50 mM SRB in 1 x PBS) and vortexed to fully dissolve. After 30 min of sonication, the vesicles were equilibrating at 4°C overnight. The next morning, vesicles were extruded 31 times through an Avanti Mini-Extruder with a size-specific filter (200 nm) to yield vesicles with the set diameter. The extruded vesicles were filtered with a NAP-10 column (GE Healthcare). The NAP-10 column was washed twice with 10 mL 1 x PBS, loaded with 400 µL of the extruded vesicles, and eluted with 20 mL of 1 x PBS. The fractions were collected, the vesicle size was determined via DLS, and the SRB content was quantified with a fluorescence spectrophotometer by adding 1% (v/v) solution of Triton X-100 (10 µL) to quantify the maximum amount of released fluorophores.[1]

1.11. Dye flux

To assay dye release via membrane-embedded LP and pore variants, a quartz cuvette with a path length of 10 mm (Hellma Analytics) was filled with SRB-encapsulated and purified DPhPC vesicles (120 µL, 100 µM lipid concentration). The fluorescence was monitored at 586 nm during excitation at 565 nm. After 5 min incubation, LP pore (30 µL, 1 µM) was added, and the fluorescence was monitored for 30 min of equilibration. Successful pore insertion and dye release of LP was recorded by irradiating the sample at 365 nm at an intensity of 154 µW cm^{-2} for 1 h.[1] After 60 min of monitoring SRB release by fluorescence monitoring, a 1% (v/v) solution of Triton X-100 (10 µL) was added to quantify the maximum amount of released fluorophores of lysed vesicles. The maximum fluorescent emission after lysing and the fluorescence before prior the addition of the pore and irradiation were used to calculate the percentual release rates.

1.12. Current recordings

Single-channel current recordings were carried out using a parallel recording set-up based on an integrated chip (Orbit Mini and Orbit 16, Nanion Technologies) with multielectrode-cavity-array chips (IONERA)[23]. Bilayers were formed of DPhPC dissolved in octane to a final concentration of 10 mg/mL. The electrophysiological buffer was composed of 1 M KCl, 10 mM HEPES, pH 7.4. For pore insertions, DNA nanopores were mixed in a 2:1 (v/v) ratio with 0.5% OPOE in 1 M KCl, 10 mM HEPES, pH 7.4. The mixture was applied to the *cis* chamber, and insertions monitored by increases in conductance steps. Current traces were acquired at 10 kHz using Element Data Recorder software (Elements). Single-channel analysis was performed using Clampfit software (Molecular Devices).

2. Supporting Tables

Table S1. Names, modifications, and sequences of DNA oligonucleotides used for the assembly of the light-controlled DNA nanopores. Asterisks indicate the position of azobenzene modifications within the sequence of the lid. Grey indicates the section which binds to the elongated hinge 1 and purple the section which binds to the elongated hinge 2. Cholesterol are attached via a tri(ethylene glycol) (TEG) linker to the 3′ terminus of strands S1-chol to S6-chol. Cyanine 5 (Cy5) is attached at the indicated internal position of S4.

ID	Sequence 5′ → 3′
S1	AGCGAACGTGGA-TTTT-GTCCGACATCGGCAAGCTCCC-TTTT-TCGACTATT
S2	CCGATGTCGGAC-TTTT-ACACGATCTTCGCCTGCTGGG-TTTT-GGGAGCTTG
S3	CGAAGATCGTGT-TTTT-CCACAGTTGATTGCCCTTCAC-TTTT-CCCAGCAGG
S4	AATCAACTGTGG-TTTT-TCTCACTGGTGATTAGAATGC-TTTT-GTGAAGGGC
S5	TCACCAGTGAGA-TTTT-TGTCGTACCAGGTGCATGGAT-TTTT-GCATTCTAA
S6	CCTGGTACGACA-TTTT-TCCACGTTCGCTAATAGTCGA-TTTT-ATCCATGCA
S2-dock	CCGATGTCGGAC-TT-CGTGCTC-TTTT-AGTCACG-TT-ACACGATCTTCGCCTGCTGGG-TTTT- GGGAGCTTG
S5-dock	TCACCAGTGAGA-TT-CGCTGCGCGGTT-TTTT-TAAGTAATCACG-TT-TGTCGTACCAGGTGCATGGAT-TTTT-GCATTCTAA
lid	GAGCACG-TTTTTTTT-CGTGATTACTTA-TTTTTTTT-AACCGCGCAGCG-TTTTTTTTT-CGTGACT
azo-lid	GA*GCA*CG-TTTTTTTT-CGTGATTACTTA-TTTTTTTT-AACCGCGCAGCG-TTTTTTTTT-CG*TGA*CT
S1-chol	S1 sequence carrying a cholesterol tri(ethylene glycol) modification at the 3′ terminus
S3-chol	S3 sequence carrying a cholesterol TEG modification at the 3′ terminus
S4-chol	S4 sequence carrying a cholesterol TEG modification at the 3′ terminus
S5-chol	S5 sequence carrying a cholesterol TEG modification at the 3′ terminus
S6-chol	S6 sequence carrying a cholesterol TEG modification at the 3′ terminus
S4-Cy5	AATCAACTGTGGTTTT/iCy5/TCTCACTGGTGATTAGAATGCTTTTGTGAAGGGC
lid-Cy5	GAGCACG-TTTTTTTT-CGTGATTACTTA-TTTTTTTT-AACCGCGCAGCG/iCy3/-TTTTTTTTT-CGTGACT

Table S2. Names and strand compositions of the light-gated nanopore LP and pore variants

Pore	Used oligonucleotides
LP-0C$_{\Delta lid}$	S1, S2, S3, S4, S5, S6
LP$_{\Delta lid}$	S1-chol, S2-dock, S3-chol, S4-chol, S5-dock, S6-chol
LP-0C$_{\Delta A}$	S1, S2-dock, S3, S4, S5-dock, S6, lid
LP-1C$_{\Delta A}$	S1-chol, S2-dock, S3, S4, S5, S6, lid
LP-2C$_{\Delta A}$	S1-chol, S2-dock, S3, S4-chol, S5, S6, lid
LP-3C$_{\Delta A}$	S1-chol, S2-dock, S3, S4-chol, S5, S6-chol, lid
LP$_{\Delta A}$	S1-chol, S2-dock, S3-chol, S4-chol, S5-dock, S6-chol, lid
LP	S1-chol, S2-dock, S3-chol, S4-chol, S5-dock, S6-chol, azo-lid
LP$_{\lambda}$	LP irradiated at 365 nm
Cy5-LP-0C$_{\Delta A}$	S1, S2-dock, S3, S4-Cy5, S5-dock, S6, lid
Cy5-LP-1C$_{\Delta A}$	S1-chol, S2-dock, S3, S4-Cy5, S5-dock, S6, lid
Cy5-LP-2C$_{\Delta A}$	S1-chol, S2-dock, S3-chol, S4-Cy5, S5-dock, S6, lid
Cy5-LP$_{\Delta A}$	S1-chol, S2-dock, S3-chol, S4-chol, S5-dock, S6-chol, lid-Cy5

3. Supporting figures

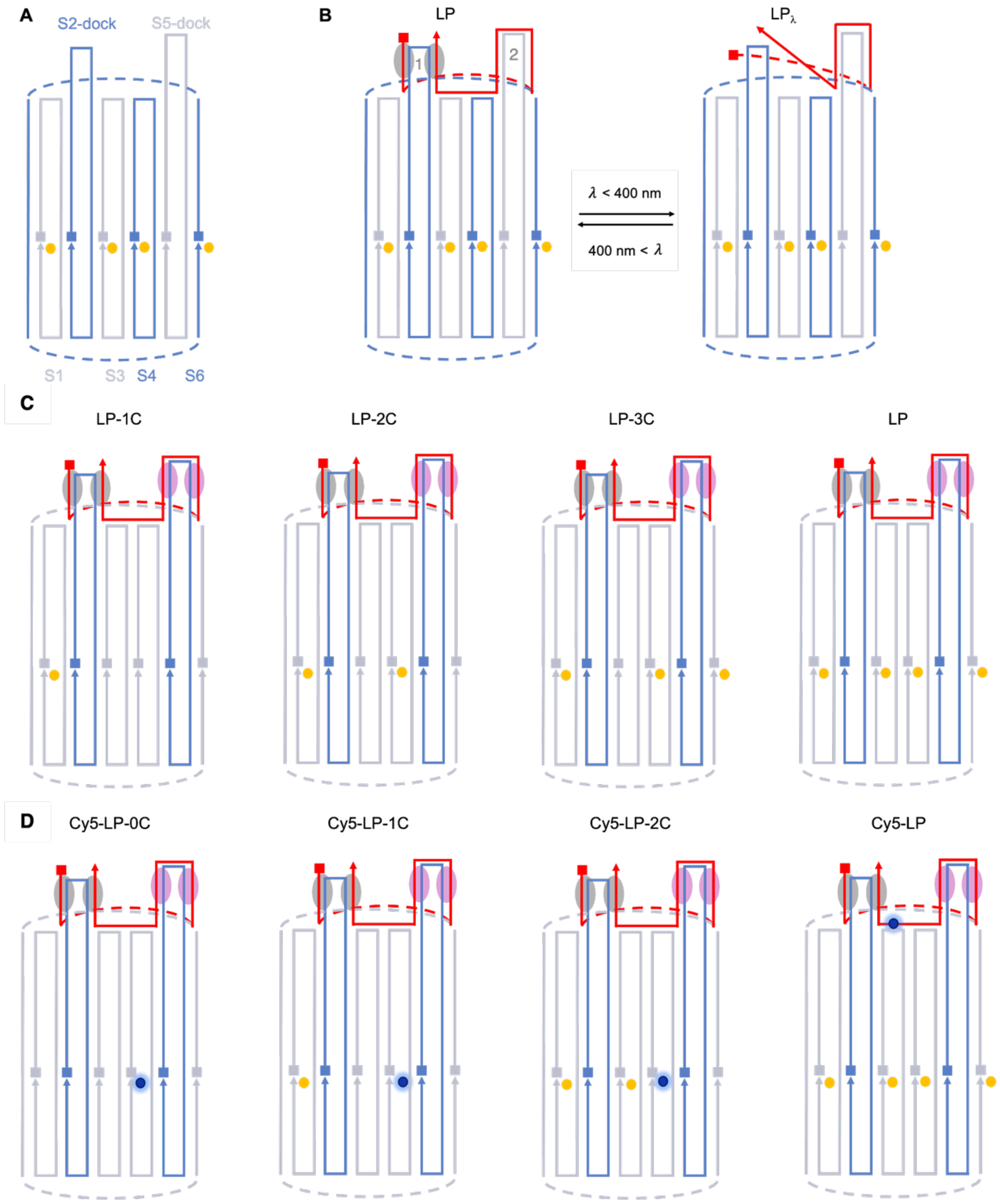

Figure S1: 2D connectivity maps of LP and variant pores. (A) 2D connectivity map showing the interaction of DNA strands in the assembled $LP_{\Delta lid}$ pore. (B) Schematic mechanism on LP's reversible lid opening upon irradiation and azobenzene isomerisation. The elongated region of the S5-dock strand (purple) holds the lid (red) in place while light-triggered isomerisation of azobenzene induces duplex breaks in the region of the S2-dock strand (grey). (C) 2D maps of light-controlled nanopores carrying 0, 1, 2 and 4 cholesterol tags. The orange dot represents the cholesterol modification attached to the 3′ terminus via a TEG linker. (D) 2D maps of fluorescence-tagged light-controlled nanopores indicating the Cy5 modification by a blue glowing dot. Squares and arrows represent 5′ and 3′ termini of oligonucleotides, respectively.

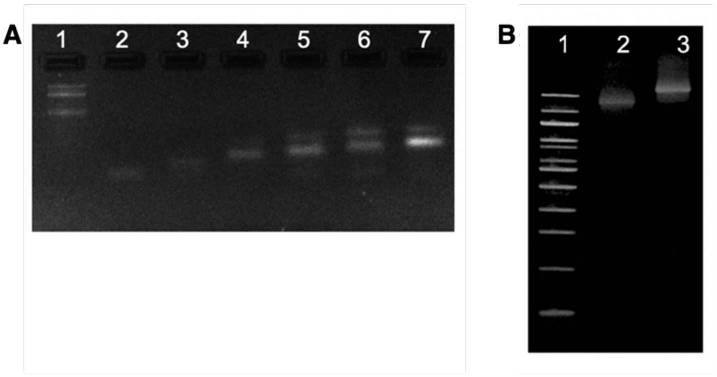

Figure S2. Gel electrophoretic mobility shift analysis on the formation of light-controlled nanopores. (A) 2.5% Agarose gel (run at 65 V for 180 min) demonstrating stepwise assembly of LP-0C$_{\Delta Lid}$. (1) 100 bp ladder, (2) strand S1, (3) strands S1-2, (4) strands S1-3, (5) strands S1- 4, (6) strands S1-5, (7) LP-0C$_{\Delta lid}$. (B) 10% 1x TGS PAGE gel (run at 110 V for 90 min) confirming incorporation of the azobenzene-modified lid strand and pore assembly of LP-0C. (1) 100 bp ladder, (2) LP-0C$_{\Delta lid}$, (3) LP-0C harbouring an azobenzene-modified lid.

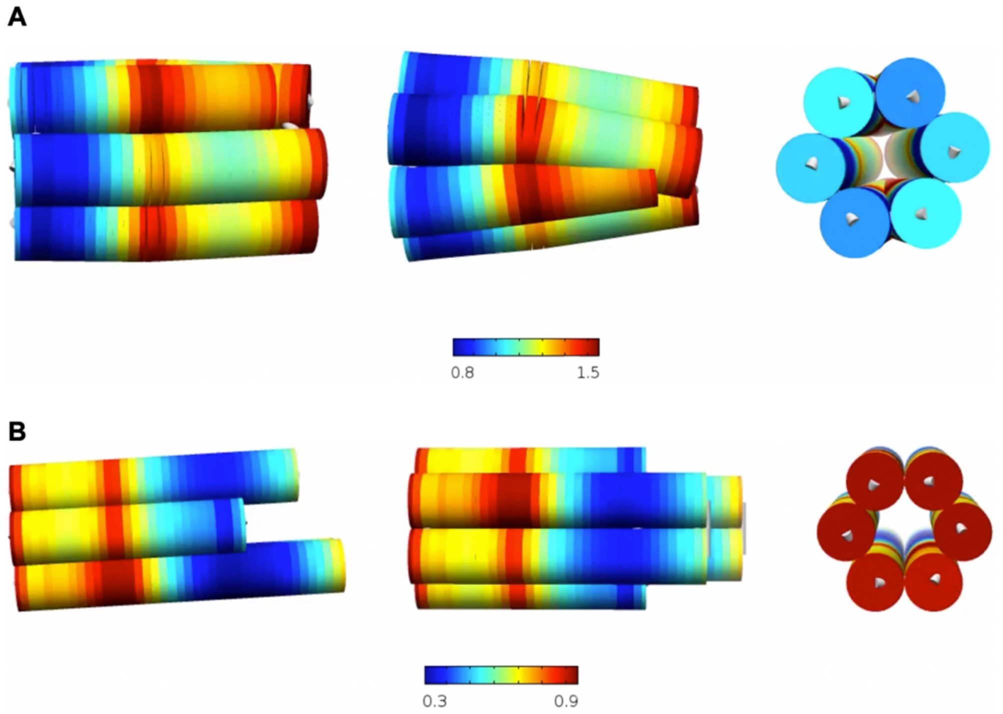

Figure S3. Thermal fluctuation analysis of LP-0C without and with lid strand. (A) Thermal fluctuation analysis of LP-0C$_{\Delta lid}$ shows a high degree of structural flexibility with RMSF values ranging from 0.8 to 1.5 nm. Lower RMSF values indicate high stability while increasing values suggest high structural fluctuation. (B) Thermal fluctuation analysis of LP-0C with the incorporated lid indicates a high stability with a max. RMSF value of 0.9 nm. The thermal fluctuation analysis was carried out on the Cando server.[4]

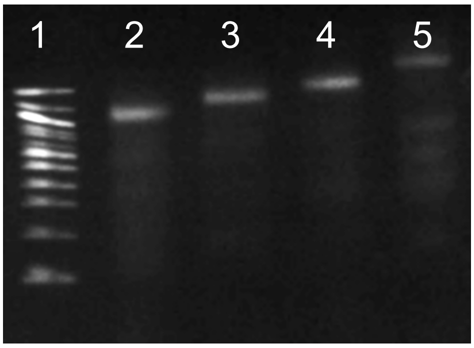

Figure S4. Gel electrophoretic mobility shift analysis of nanopores carrying up to four cholesterol anchors. 10% TGS PAGE gel analysis of (1) 100 bp ladder, (2) LP-0C$_{\Delta A}$, (3) LP-1C$_{\Delta A}$, (4) LP-2C$_{\Delta A}$, and (5) LP-4C$_{\Delta A}$.

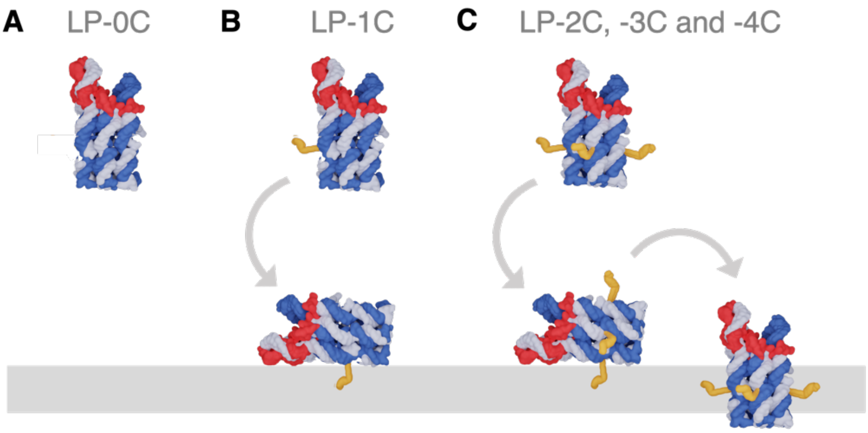

Figure S5. Scheme of membrane interaction of LP-variants in dependence of the number of cholesterol membrane anchors. (A) No binding occurs for the LC-0C without any cholesterol modification. (B) The LP-1C pore tethers sideways to the membrane. (C) LP-2C, LP-3C and LP-4C interact with the membrane via a two-step process including tethering followed by membrane puncturing.

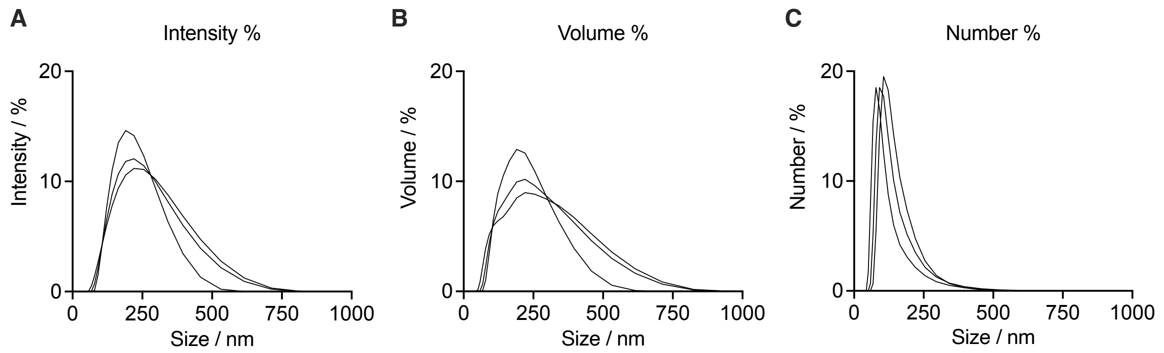

Figure S6. DLS analysis of SUVs composed of DOPC. The analysis shows the percentual (A) intensity, (B) volume, and (C) number distribution of vesicles formed by extrusion with a 200 nm filter.

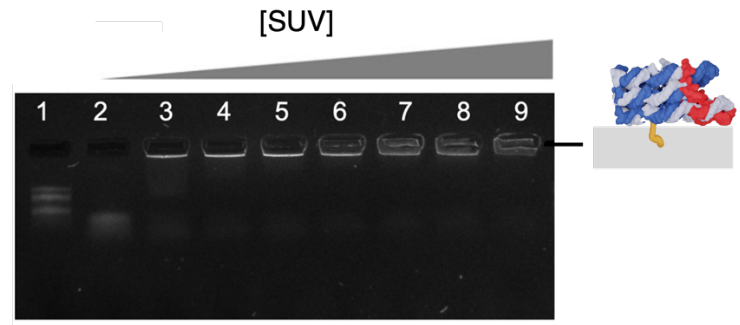

Figure S7. Gel electrophoretic analysis of LP-1C$_{\Delta A}$ binding to 200 nm DOPC vesicles. 1% agarose gel (run for 90 min at 65 V) displaying (1) a 100 bp ladder, and (2)-(9) LP-1C$_{\Delta A}$ (3 µL, 1µM) incubated with DOPC (1:1 volume ratio) vesicles at lipid concentrations ranging from 0-10 nM. An upshifted gel band indicates binding of LP-1C$_{\Delta A}$ to vesicles, as schematically illustrated.

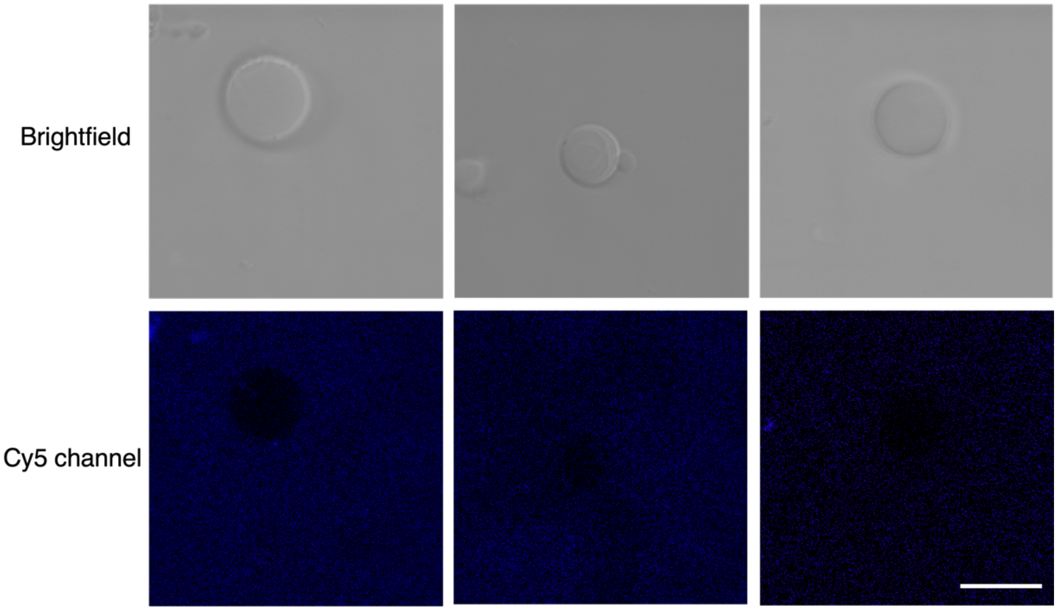

Figure S8. Confocal microscopy images on cholesterol-free Cy5-LP-0C$_{\Delta A}$ that does not bind to GUV membranes. A confocal microscope with 96x oil objective was used. Top row: brightfield images of DOPC GUVs incubated with Cy5-LP-0C$_{\Delta A}$. Bottom row: Cy5 channel images of the same GUVs showing the absence of any fluorescence signal. Scalebar, 30 μm.

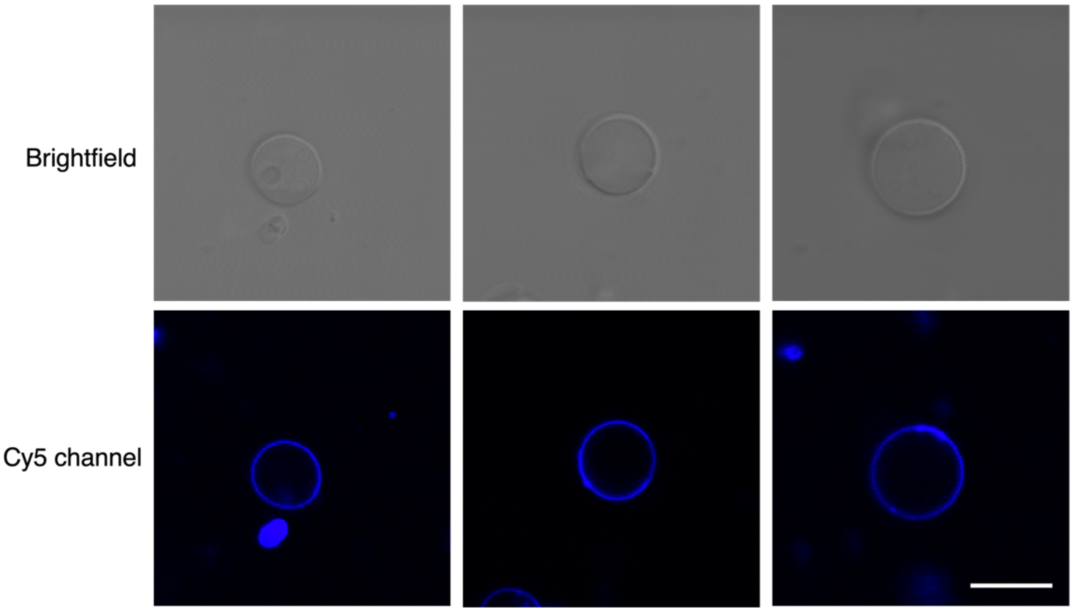

Figure S9. Confocal microscopy images confirming membrane anchoring of cholesterol-tagged Cy-5-LP-1C$_{\Delta A}$ to DOPC GUVs. A confocal microscope with 96x oil objective was used. Top row: brightfield images of DOPC GUVs incubated with Cy5-LP-1C$_{\Delta A}$. Bottom row: Cy5 channel images of the same GUVs. The fluorescence rings indicate tethering of Cy5-LP-1C$_{\Delta A}$ to GUV membranes. Scalebar, 30 μm.

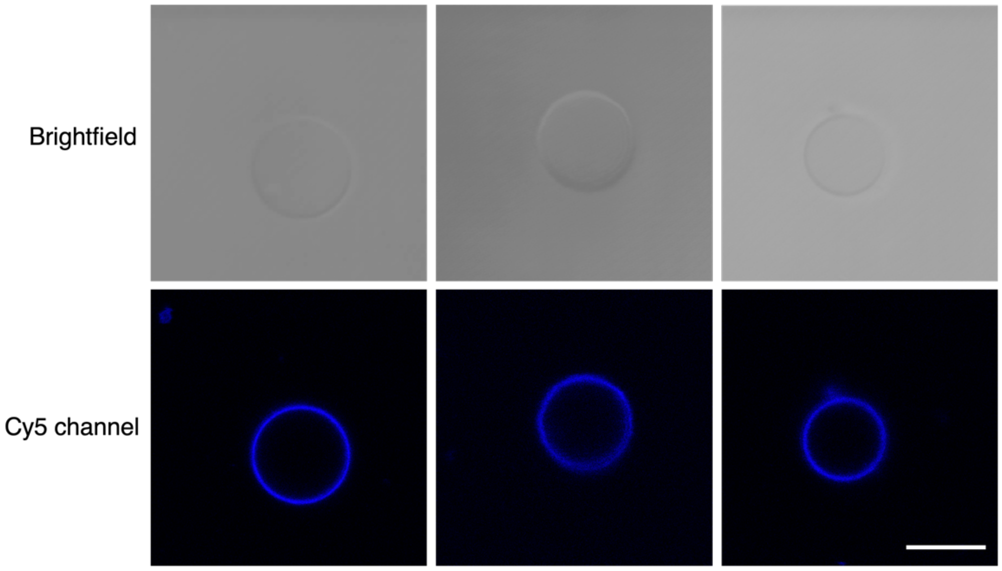

Figure S10. Confocal microscopy images confirm membrane anchoring of fluorophore-tagged Cy5-LP-2C$_{\Delta A}$ to DOPC GUV membranes. A confocal microscope with 96x oil objective was used. Top row: brightfield images of DOPC GUVs incubated with Cy5-LP-2C$_{\Delta A}$. Bottom row: Cy5 channel images of the same GUVs. The fluorescent rings indicate binding of Cy5-LP-2C$_{\Delta A}$ to the GUV membranes. Scalebar, 30 µm.

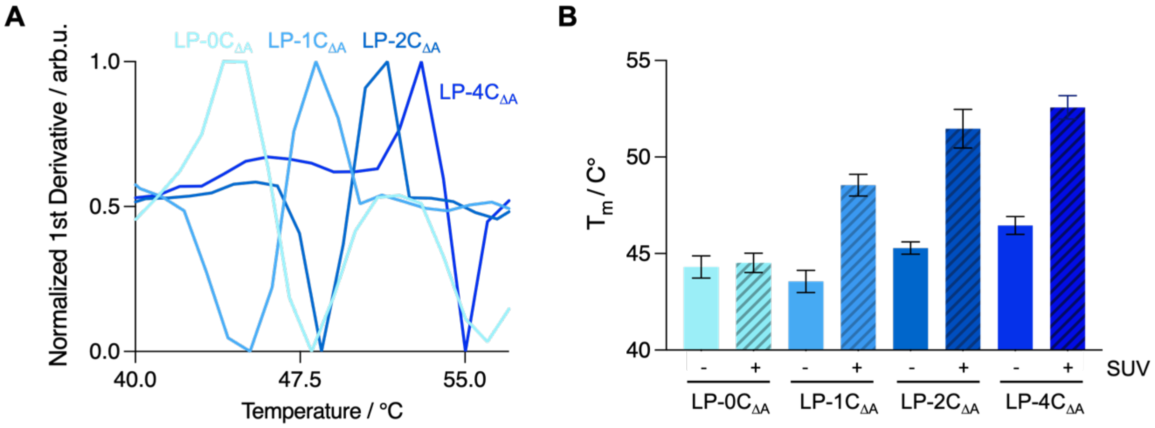

Figure S11. Membrane interaction of cholesterol-modified LP nanopores leads to their thermal stabilisation as assessed by UV melting profiles. (A) Normalised 1st derivates of UV melting curves recorded at 260 nm for LP-0C$_{\Delta A}$, LP-1C$_{\Delta A}$, LP-2C$_{\Delta A}$, and LP-4C$_{\Delta A}$ in the absence or presence of DPhPC vesicles. (B) Statistical analysis of melting temperatures for LP$_{\Delta A}$ with 0-4 cholesterol tags in the absence (non-dashed) and presence (dashed) of DPhPC SUVs (n = 3). Membrane binding and insertion is indicated by the increase in T_m by 5.5 ± 0.6 °C for LP-1C$_{\Delta A}$, 6.1 ± 1.0°C for LP-2C$_{\Delta A}$ and 6.2 ± 0.6°C for LP-4C$_{\Delta A}$ in the presence of SUV, compared to a 0.2 ± 0.5°C increase for LP-0C$_{\Delta A}$.

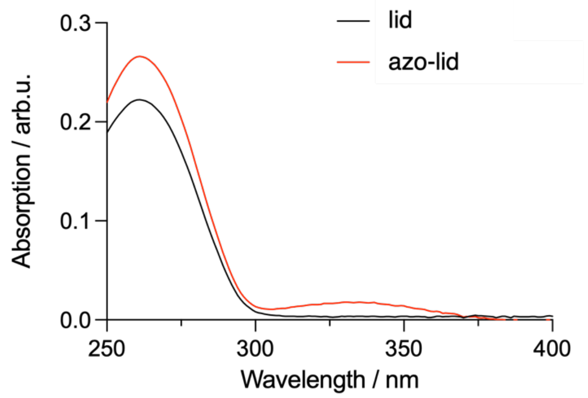

Figure S12. UV-Vis absorption spectra of the non-modified lid strand (black) and azobenzene-modified lid strand (red). Data were collected at a DNA strand concentration of 500 nM in 12 mM $MgCl_2$.

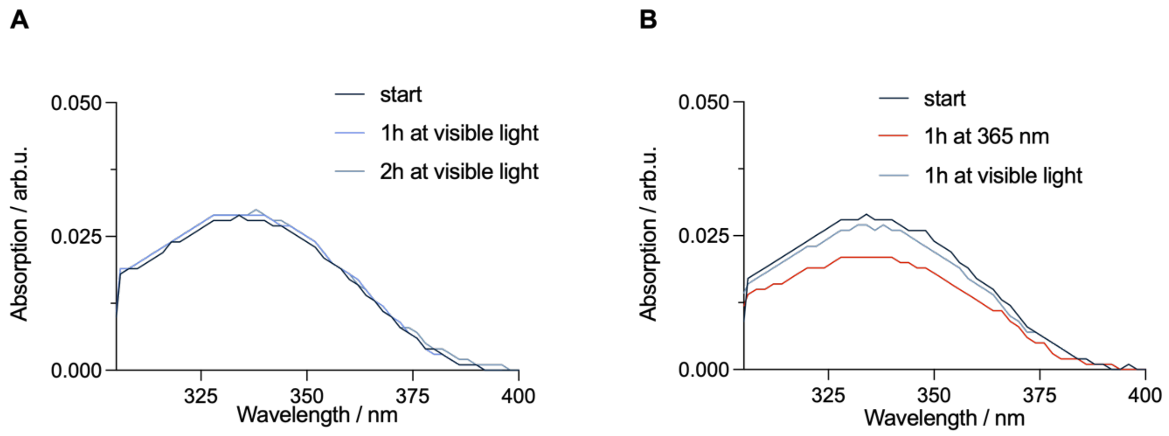

Figure S13. Reversibility of azobenzene isomerisation demonstrated by UV-Vis spectra. (A) Absorption curves of the azo-lid strand with azobenzene in *trans* form prior and up to 2 h after irradiation at visible light. No change in the spectrum is seen, as visible light maintains the chromophore in the *trans* form. (B) Absorption curves of the azo-lid strand showing the change in absorption at 340 nm after irradiation at 365 nm and azobenzene isomerisation from the *trans* to *cis* conformation with a characteristic decrease absorption at 340 nm. Exposure of the structure to visible light (390-750 nm) for 1 h leads to transition back to the *trans* conformation, shown by an increase to initial absorption at 340 nm.

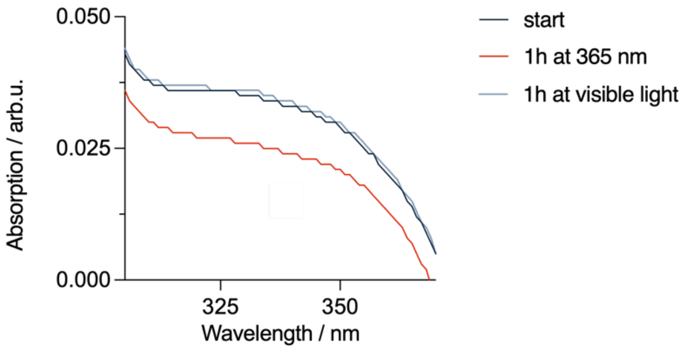

Figure S14. Reversibility of azobenzene isomerisation in the LP pore as shown with UV-Vis spectroscopy. The spectra plot the absorption curves of the azobenzene-modified pore before and after irradiation at 365 nm for 1 h, leading to a characteristic decrease in absorption at 340 nm due to the isomerisation of azobenzene from the *trans* to *cis* form. Illumination for 1 h under visible light (> 400 nm) restores the spectrum to the initial state due to isomerisation to the *trans* form. The spectra differ from the absorption readings in Figure S13 due the higher amount of nucleic acids.

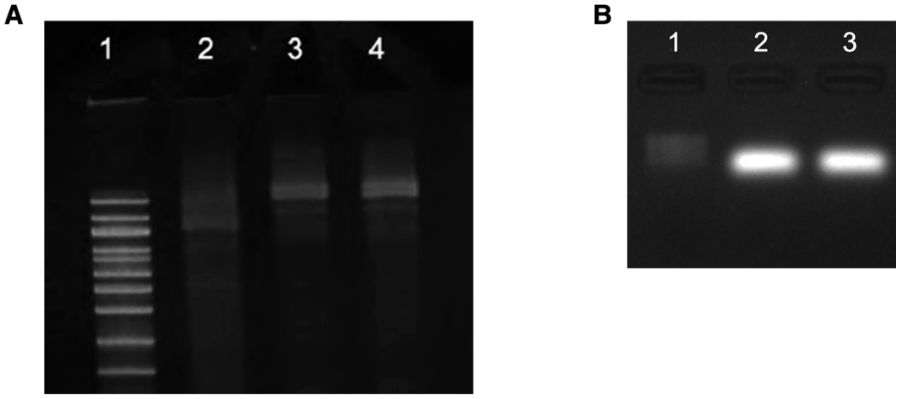

Figure S15. Structural stability of LP variants upon irradiation. The structural integrity of LP-0C$_{\Delta Lid}$, non-irradiated LP-0C and LP-0C$_{\lambda}$. (A) 10% PAGE gel (run at 100 V for 60 min) showing (1) a 100 bp ladder, (2) LP-0C$_{\Delta Lid}$, (3) LP-0C, and (4) LP-0C$_{\lambda}$ after irradiation at 365 nm for 1 h at 154 µW cm^{-2}. (B) 1% agarose gel (run at 65 V for 90 min) displaying (1) a 100 bp ladder, (2) non-irradiated LP-0C, (3) and LP-0C$_{\lambda}$ after irradiation at 365 nm for 1 h at 154 µW cm^{-2}).

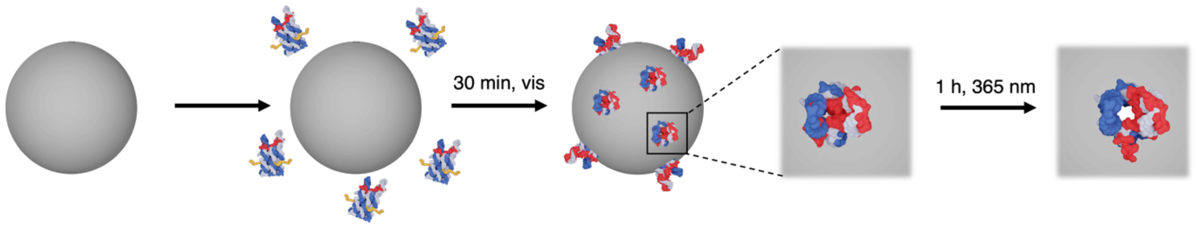

Figure S16. Scheme illustrating steps the formation of vesicles for the dye release assay. LP was inserted into small unilamellar vesicles by incubation for 30 min in visible light. Dye flux only occurs after irradiation at 365 nm and results in an opening of the azobenzene-modified lid.

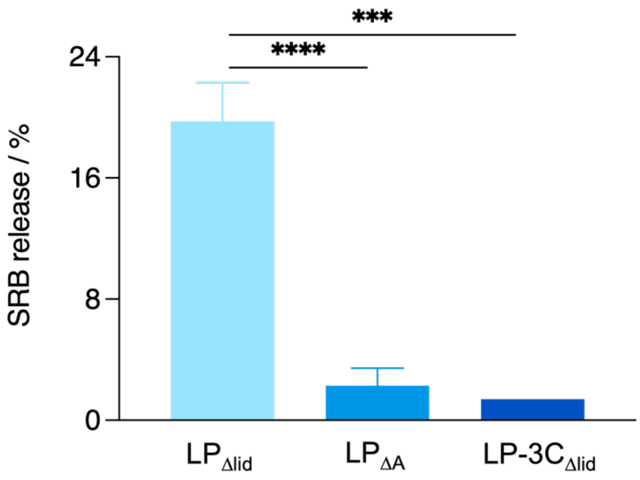

Figure S17. Dye flux from SRB-filled DPhPC vesicles is blocked by $LP_{\Delta A}$ featuring a closed lid, in comparison to the open $LP_{\Delta lid}$ pore lacking the lid. Pore $LP_{\Delta lid}$ harbouring 4 cholesterol tags inserts more efficiently into membranes when compared to LP-3C$_{\Delta lid}$ with three cholesterol anchors. A one-way ANOVA test confirms the statistically significant difference between the LP variants (n = 4).

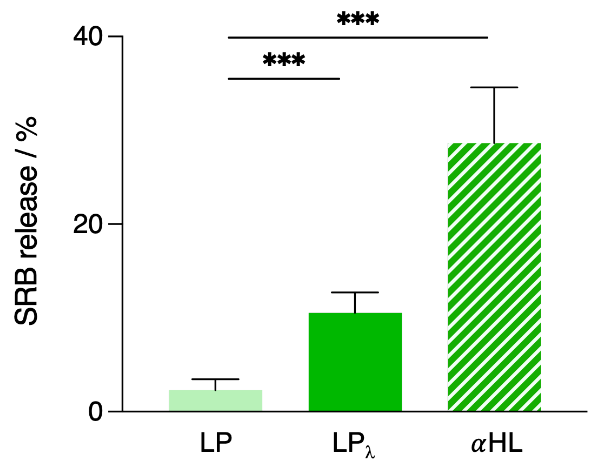

Figure S18. Dye flux from SRB-filled DPhPC vesicles incubated with LP under optional UV irradiation, in comparison to release mediated by the α-hemolysin protein nanopore. A one-way ANOVA test confirms the statistically significant differences (n = 4).

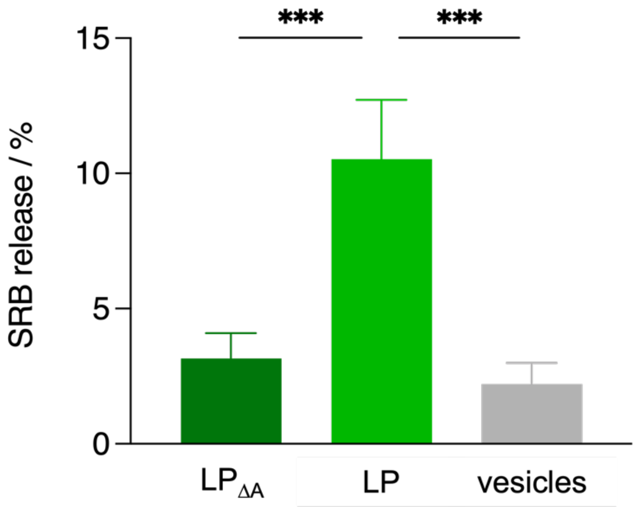

Figure S19. Dye efflux from vesicles is mediated by light-triggered opening of LP nanopores but not by any non-specific light-induced damage of DNA nanopores or membranes of vesicles. Histogram showing the dye release from SRB-filled DPhPC vesicles with inserted pore harbouring a lid without the azobenzene modifications (dark green column, LP$_{\Delta A}$), LP featuring an azobenzene-modified lid (light green column) and DPhPC vesicles without any DNA nanopore (grey column) after irradiation at 365 nm for 1 h. The rate of release for the unmodified lid is comparable to the rate for the closed pore and only slightly higher than for irradiated DPhPC vesicles.

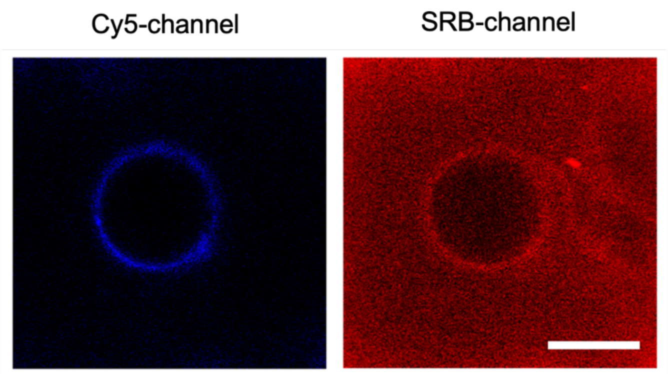

Figure S20. Confocal microscopic analysis of a DOPC GUV confirms binding of Cy5-LP$_{\Delta lid}$ to the vesicle membrane (Cy5 channel) and nanopore-mediated influx of SRB dye (SRB channel). Scalebar, 30 µm.

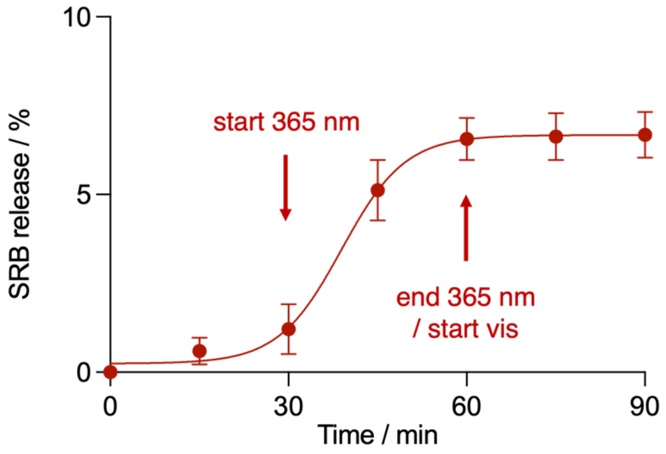

Figure S21. Reversible and light-controlled LP opening and closing, as demonstrated by kinetic analysis of SRB dye flux. SRB-filled DPhPC GUVs were incubated with LP for 30 min, followed by irradiation at 365 nm (arrow) for 30 min leading to dye efflux from SUVs via LP pores with an open lid gate. Upon exposure to visible light, dye efflux stops due to the closing of the LP gate. The datapoints were taken every 15 min and summarise five independent experiments.

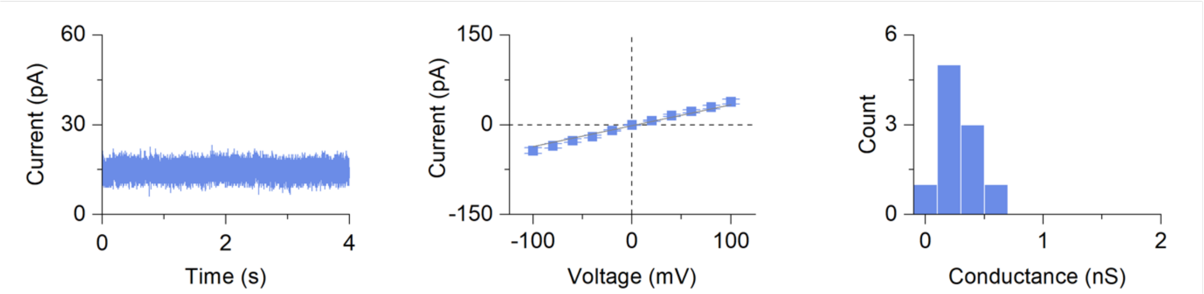

Figure S22. Single-channel current recordings of $LP_{\Delta A}$ containing a lid without an azobenzene modification, in the absence of irradiation with UV light. Analysis using representative single-channel current traces recorded at +50 mV relative to the *cis* chamber. Average current–voltage graphs (±SEM) for voltages ranging from −100 mV to +100 mV at 20 mV steps, and conductance histograms were obtained at +20 mV. For the recordings, the cholesterol-tagged nanopores were mixed with mild detergent OPOE, and the mixture was added to the *cis* chamber for membrane insertion. The recordings were acquired in 1 M KCl, 10 mM HEPES, pH 7.4.

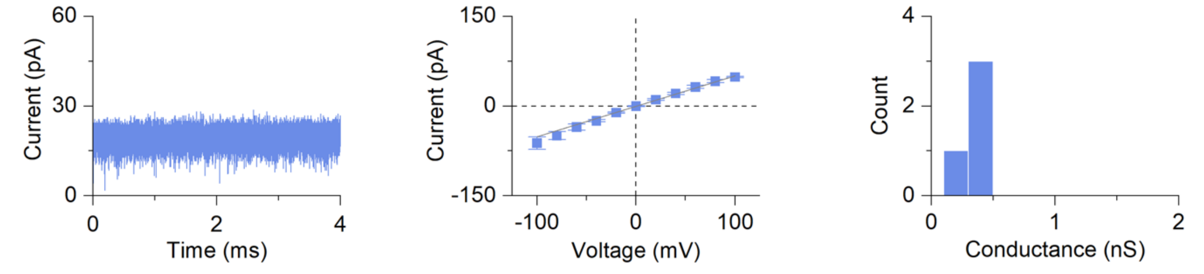

Figure S23. Single-channel current recordings of $LP_{\Delta A}$ containing a lid without an azobenzene modification, after irradiation with UV light. Analysis using representative single-channel current traces recorded at +50 mV relative to the *cis* chamber. Average current–voltage graphs (±SEM) for voltages ranging from −100 mV to +100 mV at 20 mV steps, and conductance histograms were obtained at +20 mV. For the recordings, pores were irradiated for 30 minutes at 365 nm with an intensity of 145 μW cm^{-2}. The cholesterol-tagged nanopores were mixed with mild detergent OPOE, and the mixture was added to the *cis* chamber for membrane insertion. The recordings were acquired in 1 M KCl, 10 mM HEPES, pH 7.4.

References

1 C. Lanphere, D. Offenbartl-Stiegert, A. Dorey, G. Pugh, E. Georgiou, Y. Xing, J. R. Burns and S. Howorka, *Nat. Protoc.*, 2021, **16**, 86–130.

2 Y. Xing, A. Dorey, L. Jayasinghe and S. Howorka, *Nat. Nanotechnol.*, 2022, **17**, 708–713.

3 T. Diederichs, G. Pugh, A. Dorey, Y. Xing, J. R. Burns, Q. Hung Nguyen, M. Tornow, R. Tampé and S. Howorka, *Nat. Commun.*, 2019, **10**, 5018.

4 D. N. Kim, F. Kilchherr, H. Dietz and M. Bathe, *Nucleic Acids Res.*, 2012, **40**, 2862–2868.
